# 2-Anilino-*N*-methyl-*N*-phenyl­benzamide

**DOI:** 10.1107/S1600536809013026

**Published:** 2009-04-25

**Authors:** Xing-Xing Yang, Chenglin Zhou, Da-Bin Qin

**Affiliations:** a School of Chemistry and Chemical Engineering, China West Normal University, Nanchong 637002, People’s Republic of China

## Abstract

The title compound, C_20_H_18_N_2_O, is composed of three aromatic rings, the dihedral angles between the phenyl and benzamide rings, and between the benzamide and aniline rings being 59.86 (9) and 46.57 (10)°, respectively. The mol­ecular structure is stabilized by an intra­molecular N—H⋯O hydrogen bond involving the amino H atom and the benzamide carbonyl O atom. In the crystal structure, C—H⋯O and C—H⋯π inter­actions are present.

## Related literature

For the synthesis of the title compound, see: Martín *et al.* (2006 ▶); Charton *et al.* (2006 ▶). For related structures, see: Du *et al.* (2009 ▶); Qi *et al.* (2002 ▶). For further information on mol­ecular recognition and self-assembly, see: Brunsveld *et al.* (2001 ▶); Prins *et al.* (2001 ▶).
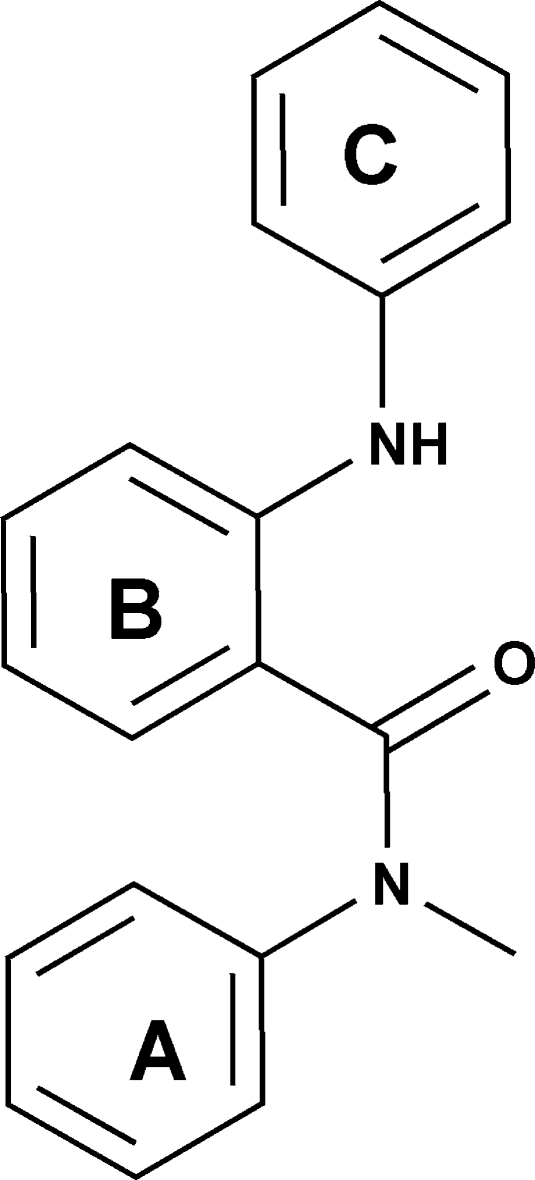


## Experimental

### 

#### Crystal data

C_20_H_18_N_2_O
*M* *_r_* = 302.36
Orthorhombic, 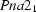

*a* = 11.086 (2) Å
*b* = 18.150 (4) Å
*c* = 7.5962 (17) Å
*V* = 1528.4 (6) Å^3^
*Z* = 4
Mo *K*α radiation
μ = 0.08 mm^−1^
*T* = 93 K
0.40 × 0.30 × 0.20 mm

#### Data collection

Rigaku Spider diffractometer
Absorption correction: none
12048 measured reflections
1887 independent reflections
1803 reflections with *I* > 2σ(*I*)
*R*_int_ = 0.034

#### Refinement

*R*[*F* ^2^ > 2σ(*F* ^2^)] = 0.033
*wR*(*F* ^2^) = 0.080
*S* = 1.18
1887 reflections
213 parameters
1 restraint
H atoms treated by a mixture of independent and constrained refinement
Δρ_max_ = 0.37 e Å^−3^
Δρ_min_ = −0.18 e Å^−3^

### 

Data collection: *RAPID-AUTO* (Rigaku/MSC, 2004 ▶); cell refinement: *RAPID-AUTO*; data reduction: *RAPID-AUTO*; program(s) used to solve structure: *SHELXS97* (Sheldrick, 2008 ▶); program(s) used to refine structure: *SHELXL97* (Sheldrick, 2008 ▶); molecular graphics: *SHELXTL* (Sheldrick, 2008 ▶); software used to prepare material for publication: *SHELXTL*.

## Supplementary Material

Crystal structure: contains datablocks global, I. DOI: 10.1107/S1600536809013026/su2103sup1.cif

Structure factors: contains datablocks I. DOI: 10.1107/S1600536809013026/su2103Isup2.hkl

Additional supplementary materials:  crystallographic information; 3D view; checkCIF report

## Figures and Tables

**Table 1:** Hydrogen-bond geometry (Å, °)

*D*—H⋯*A*	*D*—H	H⋯*A*	*D*⋯*A*	*D*—H⋯*A*
N2—H2*N*⋯O1	0.85 (2)	2.05 (2)	2.684 (2)	131.1 (18)
C9—H9⋯O1^i^	0.95	2.57	3.350 (2)	139
C2—H2⋯*Cg*2^ii^	0.95	2.75	3.420 (2)	129
C15—H15⋯*Cg*3^iii^	0.95	2.67	3.503 (2)	147
C20—H20*A*⋯*Cg*3^i^	0.98	2.67	3.441 (2)	136

Symmetry codes: (i) 
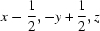
; (ii) 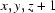; (iii) 
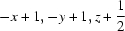
. *Cg*2 and *Cg*3 are the centroids of the C8–C13 and C14–C19 rings, respectively.

**Fig. 1.**
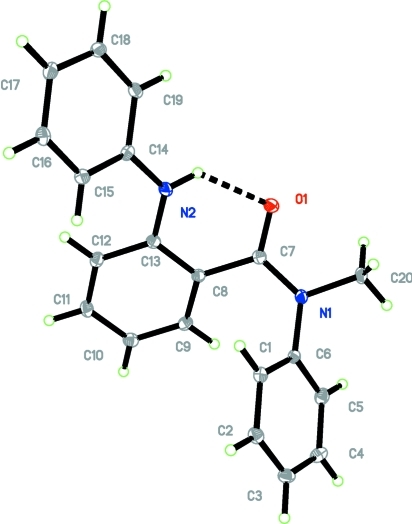
The molecular structure of the title compound, showing 30% probability displacement ellipsoids and the atomic numbering. The intramolecular N—H···O hydrogen bond is shown as a dashed line.

## supplementary crystallographic information

### Comment

Aromatic amides have found extensive applications in preorganized rationally designed monomers for efficient molecular recognition and self-assembly (Brunsveld *et al.*,  2001; Prins *et al.*,  2001). Herein we report on the crystal structure of the title compound.

The bond lengths and angles in the title compound, illustrated in Fig. 1, are within normal ranges. The dihedral angles between plane (N1/C7/O1) and rings A (C1—C6), and B (C8—C13) are 56.30 (19) and 34.59 (19)°, respectively. Rings A and C (C14—C19) are inclined to one another by 14.0 (1)°, while the central ring, B, is inclined to rings A and C by 59.86 (9) and 46.57 (10)%, respectively.

The molecular structure is stabilized by an intramolecular N—H···O hydrogen bond, involving the amino (N2) H-atom and the benzamide carbonyl O-atom (O1) (Table 1). In the crystal structure symmetry related molecules are linked by a C-H···O interaction and there are also a number of C—H···π interactions present (Table 1).

### Experimental

The title compound was prepared according to the reported procedure (Martín *et al.*, 2006); Charton *et al.*, 2006). Colourless single crystals, suitable for X-ray diffraction, were obtained by recrystallization from dichloromethane.

### Refinement

In the final cycles of refinement, in the absence of significant anomalous scattering effects, 1609 Friedel pairs were merged and Δf'' set to zero. The amino H-atom was located in a difference Fourier map and freely refined [N-H = 0.85 (2) Å]. The C-bound H atoms were placed in calculated positions [C-H = 0.95 - 0.98 Å] and treated as riding atoms [*U*_iso_(H) = 1.2 or 1.5*U*_eq_(parent C-atom)]. Friedal pairs were merged [1609 refections (85%)].

### Crystal data

**Table:**

C_20_H_18_N_2_O	*F*(000) = 640
*M**_r_* = 302.36	*D*_x_ = 1.314 Mg m^−^^3^
Orthorhombic, *P**n**a*2_1_	Mo *K*α radiation, λ = 0.71073 Å
Hall symbol: P 2c -2n	Cell parameters from 5383 reflections
*a* = 11.086 (2) Å	θ = 3.3–27.5°
*b* = 18.150 (4) Å	µ = 0.08 mm^−^^1^
*c* = 7.5962 (17) Å	*T* = 93 K
*V* = 1528.4 (6) Å^3^	Block, colorless
*Z* = 4	0.40 × 0.30 × 0.20 mm

### Data collection

**Table:**

Rigaku Spider diffractometer	1803 reflections with *I* > 2σ(*I*)
Radiation source: fine-focus sealed tube	*R*_int_ = 0.034
graphite	θ_max_ = 27.5°, θ_min_ = 3.4°
ω scans	*h* = −14→14
12048 measured reflections	*k* = −23→20
1887 independent reflections	*l* = −9→9

### Refinement

**Table:**

Refinement on *F*^2^	Primary atom site location: structure-invariant direct methods
Least-squares matrix: full	Secondary atom site location: difference Fourier map
*R*[*F*^2^ > 2σ(*F*^2^)] = 0.033	Hydrogen site location: inferred from neighbouring sites
*wR*(*F*^2^) = 0.080	H atoms treated by a mixture of independent and constrained refinement
*S* = 1.18	*w* = 1/[σ^2^(*F*_o_^2^) + (0.044*P*)^2^] where *P* = (*F*_o_^2^ + 2*F*_c_^2^)/3
1887 reflections	(Δ/σ)_max_ = 0.029
213 parameters	Δρ_max_ = 0.37 e Å^−^^3^
1 restraint	Δρ_min_ = −0.18 e Å^−^^3^

### Special details

**Table:**

Geometry. Bond distances, angles etc. have been calculated using the rounded fractional coordinates. All su's are estimated from the variances of the (full) variance-covariance matrix. The cell esds are taken into account in the estimation of distances, angles and torsion angles
Refinement. Refinement of F^2^ against ALL reflections. The weighted R-factor wR and goodness of fit S are based on F^2^, conventional R-factors R are based on F, with F set to zero for negative F^2^. The threshold expression of F^2^ > 2sigma(F^2^) is used only for calculating R-factors(gt) etc. and is not relevant to the choice of reflections for refinement. R-factors based on F^2^ are statistically about twice as large as those based on F, and R- factors based on ALL data will be even larger.

### Fractional atomic coordinates and isotropic or equivalent isotropic displacement parameters (Å^2^)

**Table:**

	*x*	*y*	*z*	*U*_iso_*/*U*_eq_	
O1	0.31344 (11)	0.23042 (7)	0.31485 (19)	0.0223 (4)	
N1	0.17863 (14)	0.22432 (8)	0.5355 (2)	0.0181 (4)	
N2	0.39961 (14)	0.36734 (8)	0.2705 (2)	0.0209 (5)	
C1	0.15046 (16)	0.31901 (10)	0.7624 (3)	0.0185 (5)	
C2	0.08545 (17)	0.34772 (10)	0.9013 (3)	0.0214 (5)	
C3	−0.02095 (17)	0.31422 (11)	0.9550 (3)	0.0227 (5)	
C4	−0.06272 (17)	0.25292 (10)	0.8662 (3)	0.0236 (5)	
C5	0.00131 (18)	0.22422 (10)	0.7240 (3)	0.0221 (5)	
C6	0.10850 (17)	0.25700 (10)	0.6736 (2)	0.0168 (5)	
C7	0.23363 (16)	0.26105 (9)	0.4024 (2)	0.0170 (5)	
C8	0.19527 (16)	0.33817 (9)	0.3594 (2)	0.0167 (5)	
C9	0.07452 (17)	0.35920 (9)	0.3734 (3)	0.0186 (5)	
C10	0.03721 (17)	0.42992 (10)	0.3310 (3)	0.0211 (5)	
C11	0.12207 (17)	0.48030 (10)	0.2705 (2)	0.0210 (5)	
C12	0.24121 (17)	0.46011 (9)	0.2502 (3)	0.0199 (5)	
C13	0.28079 (16)	0.38908 (10)	0.2941 (2)	0.0174 (5)	
C14	0.50151 (16)	0.41172 (10)	0.2448 (3)	0.0184 (5)	
C15	0.51552 (17)	0.47993 (9)	0.3279 (3)	0.0198 (5)	
C16	0.61982 (17)	0.52057 (10)	0.3017 (3)	0.0207 (5)	
C17	0.71193 (17)	0.49442 (10)	0.1958 (3)	0.0209 (5)	
C18	0.69897 (16)	0.42597 (10)	0.1170 (3)	0.0200 (5)	
C19	0.59525 (16)	0.38490 (10)	0.1413 (3)	0.0193 (5)	
C20	0.21883 (19)	0.14779 (10)	0.5689 (3)	0.0233 (6)	
H1	0.22390	0.34160	0.72730	0.0220*	
H2	0.11360	0.39050	0.96040	0.0260*	
H2N	0.4137 (18)	0.3218 (13)	0.255 (3)	0.030 (6)*	
H3	−0.06480	0.33330	1.05230	0.0270*	
H4	−0.13580	0.23010	0.90230	0.0280*	
H5	−0.02840	0.18250	0.66220	0.0260*	
H9	0.01670	0.32430	0.41300	0.0220*	
H10	−0.04500	0.44370	0.34300	0.0250*	
H11	0.09780	0.52910	0.24290	0.0250*	
H12	0.29740	0.49490	0.20560	0.0240*	
H15	0.45360	0.49840	0.40230	0.0240*	
H16	0.62820	0.56720	0.35740	0.0250*	
H17	0.78280	0.52280	0.17750	0.0250*	
H18	0.76210	0.40710	0.04550	0.0240*	
H19	0.58790	0.33800	0.08690	0.0230*	
H20A	0.23570	0.12330	0.45660	0.0280*	
H20B	0.15520	0.12090	0.63140	0.0280*	
H20C	0.29220	0.14850	0.64090	0.0280*	

### Atomic displacement parameters (Å^2^)

**Table:**

	*U*^11^	*U*^22^	*U*^33^	*U*^12^	*U*^13^	*U*^23^
O1	0.0217 (6)	0.0176 (6)	0.0277 (8)	0.0002 (5)	0.0058 (6)	−0.0024 (6)
N1	0.0235 (8)	0.0128 (7)	0.0179 (8)	0.0006 (6)	0.0020 (7)	−0.0003 (6)
N2	0.0190 (8)	0.0137 (7)	0.0300 (10)	−0.0008 (6)	0.0053 (7)	−0.0012 (7)
C1	0.0189 (8)	0.0175 (8)	0.0190 (9)	−0.0007 (7)	−0.0012 (8)	0.0013 (8)
C2	0.0259 (10)	0.0198 (9)	0.0185 (9)	0.0029 (7)	−0.0031 (8)	−0.0020 (8)
C3	0.0209 (9)	0.0295 (10)	0.0177 (9)	0.0076 (8)	0.0012 (8)	0.0014 (8)
C4	0.0190 (8)	0.0326 (10)	0.0193 (10)	−0.0017 (8)	0.0004 (8)	0.0039 (9)
C5	0.0258 (9)	0.0203 (9)	0.0202 (10)	−0.0043 (7)	−0.0014 (8)	0.0007 (8)
C6	0.0203 (9)	0.0162 (8)	0.0139 (9)	0.0023 (7)	−0.0004 (8)	0.0023 (7)
C7	0.0174 (8)	0.0160 (8)	0.0175 (9)	−0.0032 (7)	−0.0013 (8)	−0.0033 (7)
C8	0.0194 (9)	0.0165 (8)	0.0142 (9)	0.0001 (7)	0.0008 (7)	−0.0007 (7)
C9	0.0183 (9)	0.0220 (9)	0.0154 (9)	−0.0023 (7)	−0.0003 (7)	−0.0001 (8)
C10	0.0190 (9)	0.0241 (9)	0.0201 (10)	0.0027 (7)	−0.0012 (8)	0.0001 (8)
C11	0.0269 (10)	0.0169 (8)	0.0193 (10)	0.0025 (7)	−0.0037 (8)	0.0014 (8)
C12	0.0243 (9)	0.0173 (9)	0.0180 (10)	−0.0043 (7)	0.0004 (8)	0.0008 (8)
C13	0.0203 (8)	0.0178 (8)	0.0140 (9)	−0.0003 (7)	0.0003 (8)	−0.0033 (7)
C14	0.0199 (8)	0.0184 (8)	0.0169 (9)	−0.0013 (7)	0.0009 (8)	0.0033 (8)
C15	0.0208 (9)	0.0197 (9)	0.0189 (9)	0.0008 (7)	0.0021 (8)	0.0005 (8)
C16	0.0248 (9)	0.0162 (8)	0.0210 (10)	−0.0016 (7)	−0.0036 (8)	−0.0004 (7)
C17	0.0184 (9)	0.0207 (9)	0.0235 (10)	−0.0023 (7)	−0.0024 (8)	0.0060 (8)
C18	0.0178 (9)	0.0216 (9)	0.0207 (10)	0.0036 (7)	0.0017 (8)	0.0041 (8)
C19	0.0220 (9)	0.0176 (8)	0.0184 (9)	0.0036 (7)	−0.0002 (8)	0.0004 (8)
C20	0.0339 (11)	0.0132 (9)	0.0227 (10)	0.0012 (8)	0.0017 (9)	0.0018 (8)

### Geometric parameters (Å, °)

**Table:**

O1—C7	1.239 (2)	C15—C16	1.386 (3)
N1—C6	1.434 (2)	C16—C17	1.384 (3)
N1—C7	1.356 (2)	C17—C18	1.387 (3)
N1—C20	1.481 (2)	C18—C19	1.383 (3)
N2—C13	1.387 (2)	C1—H1	0.9500
N2—C14	1.401 (2)	C2—H2	0.9500
N2—H2N	0.85 (2)	C3—H3	0.9500
C1—C2	1.380 (3)	C4—H4	0.9500
C1—C6	1.392 (3)	C5—H5	0.9500
C2—C3	1.388 (3)	C9—H9	0.9500
C3—C4	1.381 (3)	C10—H10	0.9500
C4—C5	1.394 (3)	C11—H11	0.9500
C5—C6	1.383 (3)	C12—H12	0.9500
C7—C8	1.499 (2)	C15—H15	0.9500
C8—C9	1.396 (3)	C16—H16	0.9500
C8—C13	1.414 (2)	C17—H17	0.9500
C9—C10	1.387 (3)	C18—H18	0.9500
C10—C11	1.390 (3)	C19—H19	0.9500
C11—C12	1.379 (3)	C20—H20A	0.9800
C12—C13	1.402 (2)	C20—H20B	0.9800
C14—C19	1.391 (3)	C20—H20C	0.9800
C14—C15	1.398 (3)		
			
C6—N1—C7	125.87 (15)	C2—C1—H1	120.00
C6—N1—C20	115.20 (15)	C6—C1—H1	120.00
C7—N1—C20	116.98 (15)	C1—C2—H2	120.00
C13—N2—C14	128.34 (15)	C3—C2—H2	120.00
C14—N2—H2N	113.1 (14)	C2—C3—H3	120.00
C13—N2—H2N	118.0 (14)	C4—C3—H3	120.00
C2—C1—C6	120.08 (17)	C3—C4—H4	120.00
C1—C2—C3	120.20 (18)	C5—C4—H4	120.00
C2—C3—C4	119.6 (2)	C4—C5—H5	120.00
C3—C4—C5	120.59 (18)	C6—C5—H5	120.00
C4—C5—C6	119.44 (17)	C8—C9—H9	119.00
N1—C6—C5	119.37 (16)	C10—C9—H9	119.00
N1—C6—C1	120.51 (16)	C9—C10—H10	121.00
C1—C6—C5	120.05 (17)	C11—C10—H10	121.00
O1—C7—C8	120.32 (15)	C10—C11—H11	120.00
N1—C7—C8	119.61 (15)	C12—C11—H11	120.00
O1—C7—N1	120.06 (15)	C11—C12—H12	119.00
C9—C8—C13	119.41 (15)	C13—C12—H12	119.00
C7—C8—C9	120.71 (15)	C14—C15—H15	120.00
C7—C8—C13	119.77 (16)	C16—C15—H15	120.00
C8—C9—C10	121.43 (17)	C15—C16—H16	119.00
C9—C10—C11	118.94 (17)	C17—C16—H16	119.00
C10—C11—C12	120.68 (17)	C16—C17—H17	121.00
C11—C12—C13	121.16 (17)	C18—C17—H17	121.00
N2—C13—C8	119.76 (16)	C17—C18—H18	120.00
N2—C13—C12	121.88 (16)	C19—C18—H18	120.00
C8—C13—C12	118.33 (16)	C14—C19—H19	120.00
N2—C14—C15	122.39 (17)	C18—C19—H19	120.00
C15—C14—C19	118.82 (17)	N1—C20—H20A	109.00
N2—C14—C19	118.68 (17)	N1—C20—H20B	109.00
C14—C15—C16	119.94 (18)	N1—C20—H20C	109.00
C15—C16—C17	121.10 (18)	H20A—C20—H20B	109.00
C16—C17—C18	118.80 (17)	H20A—C20—H20C	109.00
C17—C18—C19	120.77 (18)	H20B—C20—H20C	109.00
C14—C19—C18	120.53 (18)		
			
C7—N1—C6—C1	−46.1 (3)	N1—C7—C8—C9	−35.8 (2)
C7—N1—C6—C5	137.06 (19)	N1—C7—C8—C13	147.89 (16)
C20—N1—C6—C1	117.39 (19)	C7—C8—C9—C10	−178.79 (18)
C20—N1—C6—C5	−59.4 (2)	C13—C8—C9—C10	−2.5 (3)
C6—N1—C7—O1	163.40 (16)	C7—C8—C13—N2	−0.1 (2)
C6—N1—C7—C8	−18.0 (3)	C7—C8—C13—C12	178.12 (16)
C20—N1—C7—O1	0.1 (2)	C9—C8—C13—N2	−176.40 (16)
C20—N1—C7—C8	178.73 (15)	C9—C8—C13—C12	1.8 (2)
C14—N2—C13—C8	−164.91 (18)	C8—C9—C10—C11	1.1 (3)
C14—N2—C13—C12	17.0 (3)	C9—C10—C11—C12	1.0 (3)
C13—N2—C14—C15	35.8 (3)	C10—C11—C12—C13	−1.7 (3)
C13—N2—C14—C19	−148.16 (19)	C11—C12—C13—N2	178.41 (17)
C6—C1—C2—C3	1.1 (3)	C11—C12—C13—C8	0.3 (3)
C2—C1—C6—N1	−176.53 (17)	N2—C14—C15—C16	178.11 (19)
C2—C1—C6—C5	0.3 (3)	C19—C14—C15—C16	2.1 (3)
C1—C2—C3—C4	−1.4 (3)	N2—C14—C19—C18	−178.01 (19)
C2—C3—C4—C5	0.4 (3)	C15—C14—C19—C18	−1.9 (3)
C3—C4—C5—C6	1.0 (3)	C14—C15—C16—C17	−0.8 (3)
C4—C5—C6—N1	175.56 (17)	C15—C16—C17—C18	−0.7 (3)
C4—C5—C6—C1	−1.3 (3)	C16—C17—C18—C19	1.0 (3)
O1—C7—C8—C9	142.75 (18)	C17—C18—C19—C14	0.3 (3)
O1—C7—C8—C13	−33.5 (2)		

### Hydrogen-bond geometry (Å, °)

**Table:**

*D*—H···*A*	*D*—H	H···*A*	*D*···*A*	*D*—H···*A*
N2—H2N···O1	0.85 (2)	2.05 (2)	2.684 (2)	131.1 (18)
C9—H9···O1^i^	0.95	2.57	3.350 (2)	139
C2—H2···Cg2^ii^	0.95	2.75	3.420 (2)	129
C15—H15···Cg3^iii^	0.95	2.67	3.503 (2)	147
C20—H20A···Cg3^i^	0.98	2.67	3.441 (2)	136

Symmetry codes: (i) *x*−1/2, −*y*+1/2, *z*; (ii) *x*, *y*, *z*+1; (iii) −*x*+1, −*y*+1, *z*+1/2.
